# Association of kidney disease index with all‐cause and cardiovascular mortality among individuals with hypertension

**DOI:** 10.1002/clc.24131

**Published:** 2023-08-21

**Authors:** Suxia Fang, Yuwen Chen, Qiyue Gao, Qucheng Wei

**Affiliations:** ^1^ Department of Cardiology, Linping Campus, Second Affiliated Hospital Zhejiang University School of Medicine Hangzhou China; ^2^ Department of Cardiology, Second Affiliated Hospital Zhejiang University School of Medicine Hangzhou China

**Keywords:** albuminuria, cardiovascular outcomes, glomerular filtration rate, hypertension, risk factor

## Abstract

**Background:**

This study aimed to investigate the association between a novel kidney disease index (KDI), which combines information from both estimated glomerular filtration rate (eGFR) and urinary albumin‐to‐creatinine ratio (uACR), and all‐cause and cardiovascular disease (CVD) mortality among individuals with hypertension.

**Methods:**

We analyzed data from 19 988 adults with hypertension who participated in the National Health and Nutrition Examination Survey from 1999 to 2018. Mortality outcomes were determined by linking to National Death Index records through December 31, 2019. Cox proportional hazards models were used to estimate hazard ratios and 95% confidence intervals for all‐cause and CVD mortality.

**Results:**

Baseline KDI levels were positively associated with glucose, insulin resistance, hemoglobin A1c, triglycerides, and C‐reactive protein (*p* value for trend <.05). During a follow‐up period of 179 859 person‐years, a total of 5069 deaths were documented, including 1741 from cardiovascular causes. After multivariable adjustment, each standard deviation increment in KDI level was associated with a 27% increased risk of all‐cause mortality and a 31% increased risk of cardiovascular deaths (both *p* < .05). Further analysis showed a J‐shaped association between KDI and mortality, with the risk increasing dramatically when KDI exceeded 0.27.

**Conclusion:**

Elevated KDI levels were significantly associated with increased mortality from all causes and CVD among individuals with hypertension. We recommend routine testing of eGFR and uACR in hypertensive patients, and using KDI as a tool to identify individuals who are most likely to benefit from preventive therapies.

**Abbreviations**
ASCVD: atherosclerotic cardiovascular disease
CKD: chronic kidney disease
CRP: C‐reactive protein
CVD: cardiovascular disease
eGFR: estimated glomerular filtration rate
HDL: high‐density lipoprotein
HOMA‐IR: homeostatic model assessment of insulin resistance
HTN: hypertension
KDI: kidney disease index
LDL: low‐density lipoprotein
NHANES: National Health and Nutrition Examination Survey
RAAS: renin–angiotensin–aldosterone system
TC: total cholesterol
TG: total triglyceride
uACR: urine albumin‐to‐creatinine ratio

## 1 INTRODUCTION

Hypertension (HTN) is a highly prevalent disorder that affects a vast majority of adults worldwide during their lifetimes. The global prevalence of HTN is estimated to be 26%, and this is expected to increase to 29% by 2025, driven largely by increases in economically developing nations.^1^ HTN is an established risk factor for cardiovascular disease (CVD),^2^, ^3^ but despite a reduction in risk with a decrease in blood pressure levels, more than half of all CVD cases occur in individuals with mild HTN or normal blood pressure.

The urine albumin‐to‐creatinine ratio (uACR) and the estimated glomerular filtration rate (eGFR) are well‐known independent risk factors for CVD and mortality among individuals with HTN.^4^, ^5^ HTN is closely linked with a decline in eGFR and a greater prevalence of albuminuria. However, these outcomes are generally viewed as being independently determined by eGFR and albuminuria. Recently, a study suggested that the kidney disease index (KDI), a composite variable that collected information from both eGFR and uACR, may provide a simpler way of identifying the highestrisk individuals who are most likely to benefit from preventive therapies.^6^

Therefore, we conducted a prospective study to investigate the associations between KDI and all‐cause and CVD mortality in a nationally representative sample of adults in the United States with HTN.

## 2 METHODS

### 2.1 Study population

**Figure 1:**
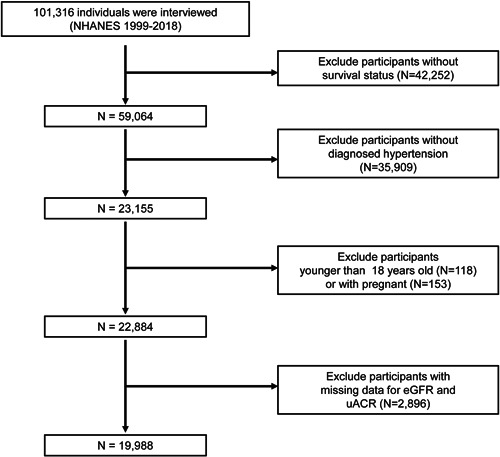
Flow diagram of the selection of eligible individuals. NHANES, National Health and Nutrition Examination Survey; uACR, urinary albumin‐to‐creatinine ratio.

The current prospective study was based on the National Health and Nutrition Examination Survey (NHANES), which is a nationwide representative survey designed to collect information on health and nutrition in the United States. Data were collected through structured interviews at home and mobile center. The entire study was approved by the Centers for Disease Control and Prevention's Institutional Review Board. Informed permission was obtained from all participants. Detailed information on the survey's design and methodology can be found on the NHANES website (https://www.cdc.gov/nchs/nhanes). In all, 101 316 participants were included in 10 consecutive circles from 1999 to 2018. We first excluded individuals without follow‐up records (*n* = 42 252), and then participants without diagnosed HTN were excluded (*n* = 35 909). In addition, we excluded individuals aged less than 18 years old (*n* = 118) or pregnant individuals (*n* = 153). This is an illustration of our exclusion criteria. Details can be found in the flow diagram (Figure 1). Finally, we excluded participants with missing data on eGFR and uACR (*n* = 2896). A total of 19 988 were finally included in the current study. HTN was defined as meeting one of the following criteria: self‐reported doctor diagnosis of HTN, use of anti‐HTN medication, or systolic blood pressure of 140 mmHg or higher, or diastolic blood pressure of 90 mmHg or high, with more than three measurements. The entire process of data selection is presented in Figure 1.

### 2.2 Definition of KDI and classification into groups

The eGFR was calculated using the Chronic Kidney Disease Epidemiology Collaboration (CKD‐EPI) Serum Creatinine 2009 equation due to its better accuracy compared to the Modification of Diet in Renal Disease equation. KDI was the geometric mean of 1/eGFR and natural log‐transformed (uACR × 100). Furthermore, KDI was categorized into quartiles: Q1 (≤0.26), Q2 (0.26–0.29), Q3 (0.29–0.33), and Q4 (>0.33).

### 2.3 Ascertainment of mortality

All‐cause mortality was defined as death due to any cause during follow‐up according to the records of the National Death Index before December 31, 2019. CVD mortality was defined as International Classification of Diseases, Tenth Revision codes I00–I09, I11, I13, I20‐I51, or I60–I69.

### 2.4 Assessment of covariates

We included various covariates that may affect the outcome, including age, sex, ethnicity, education level, family poverty income ratio, smoking and drinking status, comorbid diseases, and antihypertensive drug use. The definition of hyperlipidemia was based on meeting at least one of the following criteria: (1) self‐reported doctor‐diagnosed hyperlipidemia; (2) total triglyceride (TG) levels ≥150 mg/dL, total cholesterol (TC) levels ≥200 mg/dL, high‐density lipoprotein (HDL) levels <40 mg/dL, or low‐density lipoprotein (LDL) levels ≥130 mg/dL; or (3) the use of antihyperlipidemic medication. Atherosclerotic cardiovascular disease (ASCVD) was defined as coronary heart disease, heart attack, angina, or stroke. Creatinine, uACR, plasma glucose, insulin, hemoglobin A1C (HbA1c), TG, TC, HDL, LDL, and C‐reactive protein (CRP) were measured at recruitment when the participants provided their blood samples. The NHANES website provided detailed procedures in collecting blood sample measurements.^7^ The homeostatic model assessment of insulin resistance (HOMA‐IR) was calculated using the method of Matthews et al.^8^

### 2.5 Statistical analysis

In light of NHANES' utilization of a complex, multistage, probability sampling methodology to identify representative participants,^9^ we factored in sample weights, clustering, and stratification in all analyses to ensure that we obtained national estimates that were fully representative. Mean (standard error) was used for descriptive continuous variables, and absolute frequencies and weighted percentages were used to report categorical variables. To compare the continuous or categorical variables across the various KDI quartile groups, we utilized either the one‐way analysis of variance test, Kruskal–Wallis *H*‐test, or *χ* ^2^ test.

We utilized multivariate Cox proportional hazards regression to analyze the relationship between KDI and all‐cause and CVD mortality, to estimate the hazard ratios (HRs) and corresponding 95% confidence intervals (CIs). The reference group for our analysis was Q1, and we assigned a median value to each category to determine if there was a linear trend.

We employed restricted cubic spline regression with four knots (5th, 35th, 65th, and 95th percentiles) and multivariable adjustment to evaluate the correlation between KDI and all‐cause and CVD mortality. We conducted a likelihood ratio test to determine nonlinearity. If nonlinearity was detected, we constructed two piecewise Cox proportional hazards regression models using the inflection point.

We further conducted stratified analyses based on age, gender, ethnicity, drinking status, BMI, HTN medication use, diabetes, hyperlipidemia, and ASCVD. Additionally, we assessed potential interactions between KDI and the various stratification factors. To ensure the robustness of our findings, we conducted a sensitivity analysis. First, we excluded participants who died within 2 years of follow‐up to reduce the potential for reverse causation bias. Second, we performed further adjustments for blood lipids, HOMA‐IR, HbA1c, and CRP. Statistical significance was defined as a two‐sided *p* value less than .05. All statistical analyses were performed using R 4.2 (R Foundation for Statistical Computing).

## 3 RESULTS

The study population consisted of 19 988 adults diagnosed with HTN, with a mean (SE) age of 56.82 (0.21) years. Of these, 9924 were males (weighted, 49.65%) and 10 064 were females (weighted, 50.35%). The weighted mean (SE) eGFR was 84.67 (0.28) mL/min/1.73 m^2^, and uACR was 63.82 (3.20) mg/g. The baseline characteristics of the study population according to KDI are presented in Table 1. Participants with higher KDI levels were more likely to be older, female, and belong to the non‐Hispanic White ethnicity. They also had higher levels of the healthy eating index (HEI), and a history of prediabetes or diabetes, hyperlipidemia, and ASCVD. Conversely, they had lower levels of BMI, education, and family income.

**Table 1:** Baseline characteristics of participants with hypertension by KDI in NHANES 1999–2018.

	KDI				
	Total	≤0.26	0.26–0.29	0.29–0.33	>0.33
Participants (no.)	19 988	4 916	5 077	5024	4971
Age, mean (SE) (years)	56.82 (0.21)	43.52 (0.22)	54.95 (0.25)	63.11 (0.25)	70.43 (0.24)
Gender					
Male	9924 (49.65)	2634 (57.37)	2532 (51.63)	2354 (43.70)	2404 (42.19)
Female	10 064 (50.35)	2282 (42.63)	2545 (48.37)	2670 (56.30)	2567 (57.81)
Ethnicity					
Non‐Hispanic White	9164 (45.85)	1675 (61.41)	2170 (71.58)	2531 (76.24)	2788 (76.54)
Non‐Hispanic Black	4904 (24.53)	1630 (18.07)	1143 (11.04)	1057 (9.88)	1074 (11.73)
Mexican American	2914 (14.58)	823 (8.35)	864 (5.82)	687 (4.13)	540 (3.65)
Others	3006 (15.04)	788 (12.17)	900 (11.56)	749 (9.75)	569 (8.09)
BMI	30.78 (0.08)	31.50 (0.14)	30.85 (0.15)	30.39 (0.14)	30.15 (0.14)
HEI, mean (SE)	50.89 (0.18)	48.80 (0.28)	50.55 (0.28)	52.77 (0.29)	52.03 (0.29)
Smoking status					
Never	9950 (50.06)	2461 (49.28)	2578 (48.42)	2506 (51.04)	2405 (49.73)
Current	3674 (18.49)	1276 (26.53)	991 (19.21)	826 (16.03)	581 (10.94)
Former	6251 (31.45)	1089 (24.19)	1497 (32.37)	1681 (32.94)	1984 (39.33)
Drinking status					
Never	2875 (15.87)	511 (8.67)	702 (11.24)	746 (12.88)	916(19.60)
Mild to moderate	6164 (34.03)	1380 (33.82)	1577 (36.44)	1693 (42.07)	1514 (37.61)
Heavy	4894 (27.02)	1786 (43.25)	1450 (35.25)	1042 (24.75)	616 (14.20)
Former	4178 (23.07)	736 (14.26)	924 (17.08)	1103 (20.31)	1415 (28.58)
Education levels					
Less than high school	6079 (30.45)	1325 (18.07)	1486 (17.29)	1525 (19.57)	1743 (25.75)
High school or equivalent	4896 (24.52)	1241 (26.19)	1214 (25.86)	1204 (25.93)	1237 (27.05)
College or above	8990 (45.03)	2349 (55.74)	2374 (56.84)	2286 (54.50)	1981 (47.20)
Family income‐poverty ratio	2.96 (0.03)	2.97 (0.04)	3.15 (0.04)	3.01 (0.04)	2.62 (0.04)
eGFR (mL/min/1.73 m^2^)	84.67 (0.28)	106.63 (0.25)	90.71 (0.24)	77.19 (0.26)	54.55 (0.28)
uACR (mg/g)	63.82 (3.20)	6.65 (0.09)	13.40 (0.45)	34.04 (2.17)	250.62 (15.94)
Antihypertensive drugs	12 754 (63.89)	2082 (41.74)	3044 (58.42)	3521 (69.24)	4107 (82.68)
Self‐reported disease					
Prediabetes	6304 (31.54)	1518 (30.52)	1699 (32.49)	1677 (33.58)	1410 (29.79)
Diabetes	5876 (29.4)	898 (14.49)	1300 (20.81)	1564 (26.48)	2114 (38.82)
Hyperlipidemia	16402 (82.06)	3668 (75.91)	4180 (83.01)	4259 (86.59)	4295 (87.70)
ASCVD	3699 (18.62)	372 (6.57)	679 (12.24)	956 (16.87)	1692 (32.30)

*Note*: Data are presented as numbers (percentages), unless otherwise specified. All estimates account for complex survey designs, and all percentages are weighted.

Abbreviations: ASCVD, atherosclerotic cardiovascular disease; BMI, body mass index (calculated as weight in kilograms divided by height in meters squared); eGFR, estimated glomerular filtration rate; HEI, healthy eating index; KDI, kidney disease index; NHANES, National Health and Nutrition Examination Survey; uACR, urinary albumin‐to‐creatinine ratio.

The least‐square means of cardiometabolic biomarkers according to KDI are presented in Table 2. Our findings indicate that higher levels of KDI were significantly associated with poorer cardiometabolic health, as evidenced by higher levels of glucose, HOMA‐IR, HbA1c, TG, and CRP (all *p* values for trend <.05). Moreover, higher levels of KDI were associated with lower baseline levels of HDL.

**Table 2:** Least‐square means of cardiometabolic markers according to KDI among participants with hypertension in NHANES 1999–2018.

	KDI				*p* Value for trend
	≤0.26	0.26–0.29	0.29–0.34	>0.33	
FBG (*n* = 9788) (mmol/L)	6.10 (0.07)	6.41 (0.06)	6.72 (0.06)	7.09 (0.07)	<.001
Insulin (*n* = 9714) (pmol/L)	84.85 (3.24)	89.79 (2.84)	103.55 (2.97)	112.12 (3.30)	<.001
HOMA‐IR (*n* = 4431)	4.02 (0.24)	4.52 (0.21)	5.61 (0.22)	6.72 (0.24)	<.001
HbA1c (*n* = 19 961)	5.84 (0.02)	6.04 (0.02)	6.16 (0.02)	6.33 (0.02)	<.001
TG (*n* = 9728) (mmol/L)	1.44 (0.04)	1.66 (0.03)	1.78 (0.03)	1.88 (0.04)	<.001
TC (*n* = 19 978) (mmol/L)	5.06 (0.02)	5.23 (0.02)	5.23 (0.02)	5.12 (0.02)	.337
HDL (*n* = 19 977) (mmol/L)	1.35 (0.01)	1.36 (0.01)	1.33 (0.01)	1.28 (0.01)	<.001
LDL (*n* = 9315) (mmol/L)	3.02 0.03)	3.08 (0.02)	3.09 (0.02)	2.95 (0.03)	<.001
CRP (*n* = 11 389) (mg/L)	0.46 (0.02)	0.56 (0.02)	0.56 (0.02)	0.67 (0.02)	<.001

*Note*: Least‐square mean (SE) was estimated using a general linear model with adjustment of age (continuous), sex (male or female), ethnicity (non‐Hispanic White, non‐Hispanic Black, Mexican American, or other), BMI (continuous), education level (less than high school, high school or equivalent, or college or above), smoking status (never smoker, current smoker, former smoker), drinking status (nondrinker, low‐to‐moderate drinker, heavy drinker, or former drinker), HEI and antihypertensive drugs (yes or no).

Abbreviations: BMI, body mass index; CRP, C‐reactive protein; FBG, fasting blood glucose; HbA1c, glycated hemoglobin A1c; HDL, high‐density lipoprotein; HEI, healthy eating index; HOMA‐IR, homeostatic model assessment of insulin resistance; KDI, kidney disease index; LDL, low‐density lipoprotein; NHANES, National Health and Nutrition Examination Survey; TC, total cholesterol; TG, total triglyceride.

**Figure 2:**
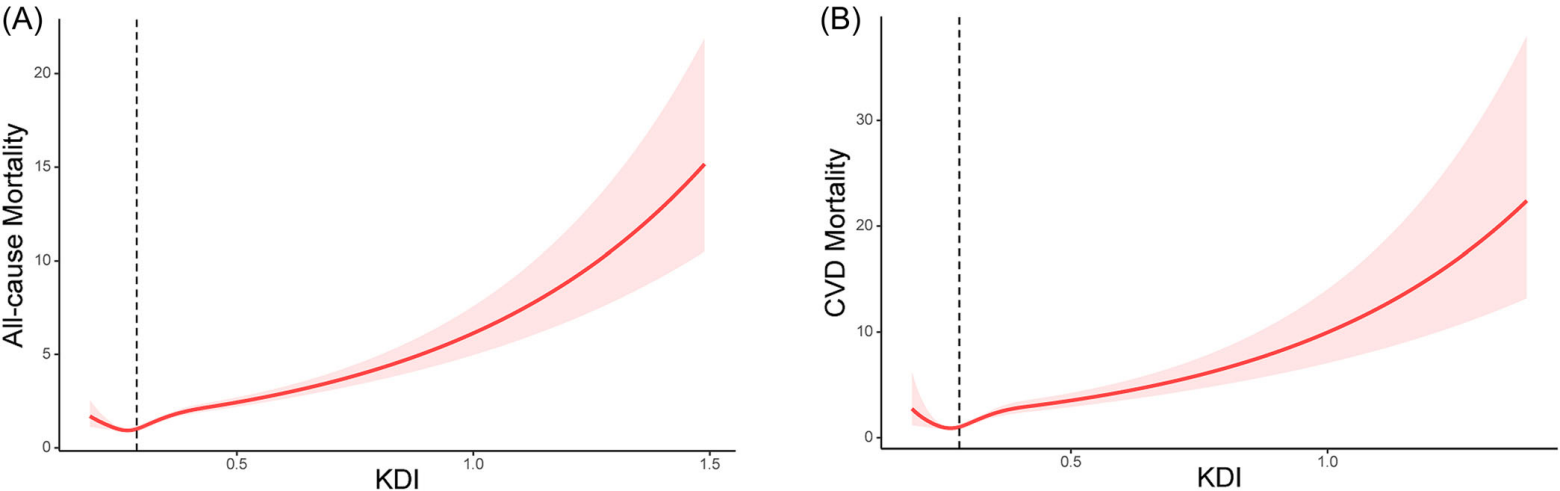
Restricted cubic spline regression for the associations between kidney disease index and all‐cause mortality (A) and cardiovascular disease mortality (B).

Over a follow‐up period of 179 859 person‐years, a total of 5069 deaths were recorded, including 1741 from cardiovascular causes. After adjusting for age, gender, ethnicity, BMI, lifestyle factors, and the presence of chronic disease, our analysis revealed that higher KDI levels were significantly associated with increased all‐cause and cardiovascular mortality. As presented in Table 3, the multivariable‐adjusted HRs and 95% CIs for all‐cause mortality, from the lowest to the highest KDI categories (≤0.26, 0.26–0.29, 0.29–0.33, and >0.33), were 1.00 (reference), 0.92 (0.77, 1.11), 1.16 (0.96, 1.40), and 1.77 (1.45, 2.15), respectively (*p* value for trend <.05). Additionally, the corresponding HRs and 95% CIs for cardiovascular death were 1.00 (reference), 0.80 (0.57, 1.14), 1.19 (0.81, 1.75), and 2.16 (1.46, 3.20), respectively (*p* value for trend <.05). Furthermore, for each standard deviation increase in KDI level, there was a 27% increased risk of all‐cause mortality and a 31% increased risk of cardiovascular deaths (Table 3). Figure 2 depicts a graphical representation of the nonlinear relationship between KDI and all‐cause and cardiovascular deaths (*p* value for nonlinearity <.05), with the inflection point at 0.27.

**Table 3:** HR (95% CIs) for all‐cause and CVD mortality according to KDI among participants with hypertension in NHANES 1999–2018.

	KDI					Per SD increment in KDI
	≤0.26	0.26–0.29	0.29–0.33	>0.33	*p*_trend_	
All‐cause mortality						
Death (no.)/total (no.)	383/4916	782/5077	1389/5024	2515/4971		
Model 1	Reference	0.95 (0.80,1.12)	1.21 (1.03,1.42)	2.11 (1.78,2.52)	<.001	1.29 (1.23,1.35)
Model 2	Reference	0.95 (0.79,1.14)	1.22 (1.01,1.46)	1.98 (1.63,2.40)	<.001	1.30 (1.25,1.34)
Model 3	Reference	0.92 (0.77,1.11)	1.16 (0.96,1.40)	1.77 (1.45,2.15)	<.001	1.27 (1.23,1.30)
CVD mortality						
Death (no.)	96	215	470	960		
Model 1	Reference	0.85 (0.61,1.17)	1.28 (0.89,1.83)	2.90 (1.99,4.21)	<.001	1.34 (1.25,1.43)
Model 2	Reference	0.84 (0.59,1.19)	1.29 (0.88,1.89)	2.59 (1.75,3.83)	<.001	1.36 (1.29,1.42)
Model 3	Reference	0.80 (0.57,1.14)	1.19 (0.81,1.75)	2.16 (1.46,3.20)	<.001	1.31 (1.26,1.37)

*Note*: Model 1—Adjusted for age (continuous), sex (male or female), and ethnicity (non‐Hispanic White, non‐Hispanic Black, Mexican American, or other). Model 2—Further adjusted for BMI (continuous), education level (less than high school, high school or equivalent, or college or above), family income–poverty ratio (continuous), smoking status (never smoker, current smoker, or former smoker), drinking status (nondrinker, low‐to‐moderate drinker, heavy drinker, or former drinker), HEI (continuous). Model 3—Further adjusted for antihypertensive drugs, diabetes or prediabetes, hyperlipidemia, and ASCVD (yes, or no).

Abbreviations: ASCVD, atherosclerotic cardiovascular disease; BMI, body mass index; CI, confidence interval; CVD, cardiovascular disease; HEI, healthy eating index; HR, hazard ratio; KDI, kidney disease index; NHANES, National Health and Nutrition Examination Survey.

The results were consistent when we conducted stratified analyses by age, sex, ethnicity, BMI, drinking status, prediabetes or diabetes, hyperlipidemia, and ASCVD (Supporting Information: Table 1). Moreover, we found no significant interactions between KDI and these stratifying variables (*p* value for interaction ≥.05).

In our sensitivity analysis, we excluded participants who died within 2 years of follow‐up (Supporting Information: Table 2), and the results remained consistent with the primary analysis. Moreover, after we adjusted for TG, HDL, LDL, HOMA‐IR, HbA1c, or CRP, there were no significant changes in the findings (Supporting Information: Tables 3–5).

## 4 DISCUSSION

We conducted a large prospective cohort study on US adults with HTN and found that an elevated KDI was significantly associated with higher mortality outcomes, including all‐cause mortality and cardiovascular deaths, independent of established risk factors such as age, gender, ethnicity, BMI, smoking or drinking status, prediabetes or diabetes, hyperlipidemia, ASCVD, and antihypertensive drug use. Stratifying our analysis by age, gender, ethnicity, BMI, antihypertensive drug use, and comorbidity showed consistent findings in each stratum. Further subgroup analysis adjusting for blood lipids, HOMA‐IR, HbA1c, and CRP did not significantly change the results. Sensitivity analysis, excluding participants who died within 2 years, yielded similar findings. Elevated KDI was also associated with higher levels of cardiometabolic biomarkers, such as glucose, HOMA‐IR, HbA1c, TG, and CRP. Restricted cubic spline analysis showed a J‐shaped association between KDI and mortality among individuals with HTN, with an inflection point of 0.27. When KDI exceeded 0.27, the risk of all‐cause and CVD mortality significantly increased. Our findings suggest that incorporating KDI into cardiovascular risk prediction can be a useful tool for identifying hypertensive patients at higher risk of mortality and initiating appropriate interventions.

HTN serves as a prevalent etiological factor for renal impairment, resulting in a decline in eGFR and an elevation in uACR. Nonetheless, prior investigations have predominantly approached eGFR and uACR as distinct entities, seldom combining them into a composite variable for comprehensive analysis. Indeed, previous studies have shown that eGFR and uACR are independent risk factors of all‐cause mortality in hypertensive patients.^10^, ^11^ Mahmoodi et al.^5^ analyzed data for 45 cohorts with 364 344 participants with HTN, and found that both eGFR and uACR were associated with increased risk of cardiovascular mortality. Matsushita et al.^12^ analyzed 637 315 individuals without a history of CVD from 24 cohorts and found similar results. Further, even microalbuminuria may indicate a high CVD risk.^13^ In the 2012 Kidney Disease Improving Global Outcomes guidelines, eGFR and urinary protein were also included as criteria for classifying CKD stages. However, few studies have investigated the interactions between these two variables or combined them as a composite variable. Furthermore, although renin–angiotensin–aldosterone system inhibitors are recommended as initial therapy for most patients with HTN, few HTN guidelines recommend universal eGFR and uACR testing in HTN.^2^ Previous meta‐analyses have demonstrated that among 20 general population cohorts, only 4.1% (ranging from 1.3% to 20.7%) of individuals underwent uACR testing during the baseline period. Furthermore, among patients with an eGFR less than 60, a mere 6.2% (ranging from 1.8%–3.17%) received uACR testing.^14^ In addition, renin–angiotensin–aldosterone system (RAAS) inhibitor use is relatively low, with only about 40% of patients with HTN taking them at baseline.^14^ Based on our findings, physicians should be aware of the potential clinical implications of decreased eGFR levels and elevated uACR levels in hypertensive patients. We recommend that eGFR and uACR be routinely tested in hypertensive patients and that clinicians consider using KDI as a composite variable derived from both eGFR and uACR to assess cardiovascular risk in hypertensive patients.

Using geometric mean to combine continuous variables into a composite variable, as suggested by Gerstein et al.,^15^ reduces the number of variables needed for analysis and combines information from different aspects into a single index. Gerstein et al.^6^ first use the KDI in type 2 diabetes patients to assess the association between KDI and CVD mortality. To our knowledge, our study was the first to simultaneously combine the information from eGFR and uACR using the KDI to assess mortality risk among hypertensive individuals. Our study demonstrates a nonlinear association between KDI and mortality, with a significant increase in all‐cause and CVD mortality risk observed when KDI exceeded 0.27. These findings suggest that KDI may serve as a useful tool for risk stratification and intervention in hypertensive patients.

Although the exact mechanisms underlying the association between eGFR and albuminuria and CVD are not fully understood, evidence from animal and human studies suggests that albuminuria is associated with abnormalities in endothelial glycocalyx and other endothelial structures, which may lead to inflammation and endothelial dysfunction.^16^, ^17^, ^18^ Furthermore, significant coronary artery stenosis has been found by angiography in about half of the predialysis patients with extremely low levels of eGFR, indicating a potential link between kidney function and atherosclerosis.^19^ However, further mechanistic studies are needed to elucidate the precise mechanisms underlying the association between eGFR and albuminuria levels and the higher risk of all‐cause and cardiovascular mortality.

Prior investigations have generated divergent findings concerning the capacity of eGFR and uACR to enhance CVD prediction beyond conventional models. Chang et al.^20^ revealed that the incorporation of eGFR and uACR into the Framingham model failed to enhance the prognostic utility of the model in evaluating CVD risk among non‐CKD patients. In contrast, Nerpin et al.^21^ posited that eGFR and uACR exhibit promising potential in refining the predictive accuracy of models grounded in traditional CVD risk factors among elderly males. Significantly, Matsushita et al.^22^ made the noteworthy observation that the augmentation of eGFR and uACR to the recently introduced CVD prediction models, namely, SCORE2 and SCORE2‐OP, developed by the European Society of Cardiology in 2021, resulted in amplified predictive efficacy for CVD. Further incorporating elevated KDI levels into cardiovascular risk prediction may serve as a useful and simple counseling point for patients.

Further research is needed to determine the optimal management strategies for patients with KDI levels exceeding 0.27, such as more intensive blood pressure targets, earlier initiation of RAAS blockade, or more frequent monitoring of kidney function. Additionally, future studies should evaluate whether interventions targeting KDI levels can improve cardiovascular outcomes in hypertensive patients.

Our study has several strengths, including large sample size, a prospective design, and comprehensive data collection. However, there are several limitations that need to be acknowledged. Firstly, the observational study design does not allow us to establish causality. Secondly, using a single measurement for KDI may not accurately reflect long‐term levels and changes over time. Thirdly, changes in covariates over time may have attenuated the true association between KDI levels and mortality. Fourthly, we did not have detailed information on the severity of HTN. Finally, the possibility of residual or unknown confounding cannot be entirely ruled out.

## 5 CONCLUSION

Our findings demonstrate a significant positive association between KDI, a composite variable derived from both eGFR and uACR, and increased risk of all‐cause and CVD mortality in hypertensive patients. Notably, we observed a J‐shaped relationship between KDI and mortality outcomes, with an incremental increase in mortality risk when KDI exceeded 0.27. These results highlight the potential clinical significance of KDI in predicting adverse outcomes, including mortality, in hypertensive patients. We recommend that eGFR and uACR be routinely tested in hypertensive patients, in addition to current HTN guidelines, and that KDI be used as a tool to identify individuals at the highest risk who are most likely to benefit from preventive therapies.

## CONFLICT OF INTEREST STATEMENT

The authors declare no conflict of interest.

## Supporting information

Supporting information.

Supporting information.

Supporting information.

Supporting information.

Supporting information.

## Data Availability

We utilized the NHANES database in our study, and the details can be accessed at: http://www.cdc.gov/nchs/nhanes/.
