# Supplementary material for: Association of kidney disease index with all‐cause and cardiovascular mortality among individuals with hypertension
Source: Clin Cardiol. 2023 Aug 21;46(11):1442–9. doi: 10.1002/clc.24131 (PMC10642315; doi:10.1002/clc.24131)
Supplement: Supplementary file 1 — Supporting information. [file CLC-46-1442-s004.docx]

**Supplementary Table 1. Stratified analyses of the associations (hazard ratios, 95% CIs) between KDI and all-cause mortality among participants with hypertension in NHANES 1999-2018**

|  | KDI | | | | | *P* interaction |
| --- | --- | --- | --- | --- | --- | --- |
|  | ≤0.26 | 0.26-0.29 | 0.29-0.33 | >0.33 | *P* trend |  |
| Age |  |  |  |  |  | 0.15 |
| ≤60 years | Reference | 1.26(1.00,1.59) | 1.97(1.45,2.67) | 3.46(2.65,4.53) | <0.001 |  |
| >60 years | Reference | 1.07(0.82,1.39) | 1.56(1.22,2.00) | 2.96(2.32,3.78) | <0.001 |  |
| Sex |  |  |  |  |  | 0.43 |
| Male | Reference | 0.93(0.73,1.18) | 1.14(0.89,1.47) | 1.80(1.38,2.34) | <0.001 |  |
| Female | Reference | 0.95(0.73,1.22) | 1.22(0.94,1.58) | 1.78(1.38,2.31) | <0.001 |  |
| Ethnicity |  |  |  |  |  | 0.66 |
| Non-Hispanic White | Reference | 0.87(0.68,1.11) | 1.06(0.82,1.36) | 1.55(1.18,2.03) | <0.001 |  |
| Other | Reference | 1.05(0.84,1.31) | 1.38(1.12,1.70) | 2.43(1.97,3.00) | <0.001 |  |
| Drinking status |  |  |  |  |  | 0.09 |
| Nondrinker | Reference | 1.14(0.82,1.58) | 1.42(1.00,2.00) | 2.22(1.59,3.11) | <0.001 |  |
| Current drinker | Reference | 0.82(0.67,1.00) | 1.04(0.84,1.28) | 1.51(1.20,1.90) | <0.001 |  |
| BMI, kg/m^2^ |  |  |  |  |  | 0.52 |
| <30 | Reference | 0.88(0.69,1.14) | 0.99(0.77,1.27) | 1.48(1.14,1.92) | <0.001 |  |
| ≥30 | Reference | 0.93(0.71,1.22) | 1.35(1.03,1.78) | 2.11(1.58,2.83) | <0.001 |  |
| Hypertension medical use |  |  |  |  |  | 0.22 |
| No | Reference | 0.87(0.66,1.15) | 1.06(0.80,1.42) | 1.40(1.00,1.98) | 0.003 |  |
| Yes | Reference | 0.96(0.74,1.26) | 1.23(0.95,1.61) | 1.95(1.51,2.52) | <0.001 |  |
| Diabetes |  |  |  |  |  | 0.83 |
| No | Reference | 0.81(0.61,1.08) | 0.96(0.72,1.28) | 1.46(1.08,1.99) | <0.001 |  |
| Prediabetes | Reference | 0.94(0.65,1.36) | 1.06(0.73,1.54) | 1.48(1.00,2.19) | <0.001 |  |
| Diabetes | Reference | 1.01(0.68,1.50) | 1.44(0.97,2.13) | 2.39(1.65,3.47) | <0.001 |  |
| Hyperlipidemia |  |  |  |  |  | 0.57 |
| No | Reference | 0.96(0.65,1.43) | 0.98(0.67,1.44) | 1.49(1.01,2.18) | <0.001 |  |
| Yes | Reference | 0.91(0.75,1.12) | 1.19(0.98,1.46) | 1.82(1.48,2.25) | <0.001 |  |
| ASCVD |  |  |  |  |  | 0.97 |
| No | Reference | 0.90(0.73,1.12) | 1.13(0.91,1.40) | 1.71(1.35,2.18) | <0.001 |  |
| Yes | Reference | 1.03(0.68,1.56) | 1.31(0.86,2.01) | 2.03(1.31,3.14) | <0.001 |  |

Abbreviations: ASCVD, atherosclerotic cardiovascular disease; BMI, body mass index; NHANES, National Health an Nutrition Examination Survey.

Adjusted for age, sex, ethnicity, BMI, education level, family income-poverty ratio, smoking status, drinking status, HEI, antihypertensive drugs, diabetes or prediabetes, hyperlipidemia, ASCVD.
