# Supplementary material for: Association of kidney disease index with all‐cause and cardiovascular mortality among individuals with hypertension
Source: Clin Cardiol. 2023 Aug 21;46(11):1442–9. doi: 10.1002/clc.24131 (PMC10642315; doi:10.1002/clc.24131)
Supplement: Supplementary file 2 — Supporting information. [file CLC-46-1442-s002.docx]

**Supplementary Table 2. Hazard ratios (95% CIs) of all-cause and CV mortality according to KDI among participants with hypertension after excluding participants who died within 2 years**

|  | KDI | | | | |
| --- | --- | --- | --- | --- | --- |
|  | ≤0.26 | 0.26-0.28 | 0.28-0.32 | >0.32 | *P*_trend_ |
| All-cause mortality |  |  |  |  |  |
| Death, No./total No. | 162/4371 | 296/4386 | 632/4444 | 1578/4386 |  |
| Model 1 | Reference | 0.80(0.62,1.02) | 1.09(0.84,1.42) | 2.15(1.65,2.79) | <0.001 |
| Model 2 | Reference | 0.88(0.67,1.16) | 1.23(0.91,1.68) | 2.22(1.66,2.98) | <0.001 |
| Model 3 | Reference | 0.87(0.66,1.15) | 1.21(0.88,1.65) | 2.05(1.52,2.77) | <0.001 |
| CVD mortality |  |  |  |  |  |
| Death, No. | 36 | 69 | 205 | 613 |  |
| Model 1 | Reference | 0.58(0.33,1.04) | 1.00(0.58,1.73) | 2.58(1.47,4.50) | <0.001 |
| Model 2 | Reference | 0.69(0.34,1.37) | 1.22(0.62,2.43) | 2.73(1.37,5.44) | <0.001 |
| Model 3 | Reference | 0.67(0.33,1.33) | 1.18(0.60,2.33) | 2.40(1.21,4.73) | <0.001 |

Model 1: adjusted for age (continuous), sex (male or female) and ethnicity (non-Hispanic white, non-Hispanic black, Mexican American, or other);

Model 2: further adjusted for BMI (continuous), education level (less than high school, high school or equivalent, or college or above), family income-poverty ratio (continuous), smoking status (never smoker, current smoker, or former smoker), drinking status (non-drinker, low-to-moderate drinker, heavy drinker, or former drinker), HEI (continuous);

Model 3: further adjusted for antihypertensive drugs, prediabetes or diabetes, hyperlipidemia, ASCVD (yes, or no).
