# Supplementary material for: Association of kidney disease index with all‐cause and cardiovascular mortality among individuals with hypertension
Source: Clin Cardiol. 2023 Aug 21;46(11):1442–9. doi: 10.1002/clc.24131 (PMC10642315; doi:10.1002/clc.24131)
Supplement: Supplementary file 3 — Supporting information. [file CLC-46-1442-s003.docx]

**Supplementary Table 3. Hazard ratios (95% CIs) of all-cause mortality and CVD mortality according to KDI among participants with hypertension with further adjustment of blood lipids**

|  | KDI | | | | |
| --- | --- | --- | --- | --- | --- |
|  | ≤0.26 | 0.26-0.29 | 0.29-0.33 | >0.33 | *P*_trend_ |
| All-cause mortality |  |  |  |  |  |
| Model 1 | Reference | 0.92(0.69,1.22) | 1.19(0.88,1.60) | 1.97(1.44,2.69) | <0.001 |
| Model 2 | Reference | 0.94(0.68,1.29) | 1.16(0.85,1.58) | 1.71(1.20,2.42) | <0.001 |
| Model 2 + TG + HDL + LDL | Reference | 0.93(0.68,1.28) | 1.15(0.84,1.57) | 1.70(1.21,2.40) | <0.001 |
| CVD mortality |  |  |  |  |  |
| Model 1 | Reference | 0.69(0.41,1.15) | 0.93(0.54,1.61) | 1.87(1.05,3.32) | <0.001 |
| Model 2 | Reference | 0.69(0.42,1.15) | 0.90(0.52,1.56) | 1.63(0.91,2.91) | <0.001 |
| Model 2 + TG + HDL + LDL | Reference | 0.68(0.41,1.12) | 0.87(0.51,1.50) | 1.58(0.90,2.79) | <0.001 |

Model 1: adjusted for age (continuous), sex (male or female) and ethnicity (non-Hispanic white, non-Hispanic black, Mexican American, or other);

Model 2: further adjusted for BMI (continuous), education level (less than high school, high school or equivalent, or college or above), family income-poverty ratio (continuous), smoking status (never smoker, current smoker, or former smoker), drinking status (non-drinker, low-to-moderate drinker, heavy drinker, or former drinker), antihypertensive drug, prediabetes or diabetes, hyperlipidemia, ASCVD (yes, or no).
