# Supplementary material for: Association of kidney disease index with all‐cause and cardiovascular mortality among individuals with hypertension
Source: Clin Cardiol. 2023 Aug 21;46(11):1442–9. doi: 10.1002/clc.24131 (PMC10642315; doi:10.1002/clc.24131)
Supplement: Supplementary file 4 — Supporting information. [file CLC-46-1442-s005.docx]

**Supplementary Table 4. Hazard atios (95% CIs) of all-cause mortality and CVD mortality according to KDI among participants with hypertension with further adjustment of HOMA-IR and HbA1c**

|  | KDI | | | | |
| --- | --- | --- | --- | --- | --- |
|  | ≤0.26 | 0.26-0.29 | 0.29-0.33 | >0.33 | *P*_trend_ |
| All-cause mortality |  |  |  |  |  |
| Model 1 | Reference | 0.98(0.78,1.23) | 1.34(1.03,1.73) | 2.23(1.71,2.92) | <0.001 |
| Model 2 | Reference | 1.00(0.76,1.30) | 1.28(0.96,1.70) | 1.91(1.41,2.59) | <0.001 |
| Model 2 + HOMA-IR + HbA1c | Reference | 0.99(0.76,1.29) | 1.25(0.94,1.67) | 1.87(1.38,2.54) | <0.001 |
| CVD mortality |  |  |  |  |  |
| Model 1 | Reference | 0.89(0.55,1.43) | 1.20(0.73,1.97) | 2.79(1.68,4.64) | <0.001 |
| Model 2 | Reference | 0.82(0.51,1.34) | 1.13(0.66,1.92) | 2.02(1.15,3.53) | <0.001 |
| Model 2 + HOMA-IR + HbA1c | Reference | 0.79(0.49,1.28) | 1.08(0.64,1.84) | 1.89(1.09,3.26) | <0.001 |

Model 1: adjusted for age (continuous), sex (male or female) and ethnicity (non-Hispanic white, non-Hispanic black, Mexican American, or other);

Model 2: further adjusted for BMI (continuous), education level (less than high school, high school or equivalent, or college or above), family income-poverty ratio (0-1.0, 1.0-3.0, or >3.0), smoking status (never smoker, current smoker, or former smoker), drinking status (non-drinker, low-to-moderate drinker, heavy drinker, or former drinker), antihypertensive drug, prediabetes or diabetes, hyperlipidemia, ASCVD (yes, or no).
