# Supplementary material for: Association of kidney disease index with all‐cause and cardiovascular mortality among individuals with hypertension
Source: Clin Cardiol. 2023 Aug 21;46(11):1442–9. doi: 10.1002/clc.24131 (PMC10642315; doi:10.1002/clc.24131)
Supplement: Supplementary file 5 — Supporting information. [file CLC-46-1442-s001.docx]

**Supplementary Table 5. Hazard ratios (95% CIs) of all-cause mortality and CVD mortality according to KDI among hypertension with further adjustment of CRP**

|  | KDI | | | | |
| --- | --- | --- | --- | --- | --- |
|  | ≤0.26 | 0.26-0.29 | 0.29-0.33 | >0.33 | *P*_trend_ |
| All-cause mortality |  |  |  |  |  |
| Model 1 | Reference | 0.98(0.81,1.19) | 1.23(1.05,1.45) | 2.13(1.76,2.58) | <0.001 |
| Model 2 | Reference | 0.95(0.77,1.17) | 1.18(0.97,1.44) | 1.80(1.44,2.25) | <0.001 |
| Model 2 + CRP | Reference | 0.93(0.76,1.15) | 1.16(0.96,1.41) | 1.74(1.40,2.17) | <0.001 |
| CVD mortality |  |  |  |  |  |
| Model 1 | Reference | 0.93(0.66,1.30) | 1.39(0.97,1.98) | 2.98(2.02,4.40) | <0.001 |
| Model 2 | Reference | 0.85(0.60,1.22) | 1.31(0.89,1.95) | 2.30(1.51,3.51) | <0.001 |
| Model 2 + CRP | Reference | 0.83(0.58,1.19) | 1.28(0.87,1.87) | 2.18(1.44,3.28) | <0.001 |

Model 1: adjusted for age (continuous), sex (male or female) and ethnicity (non-Hispanic white, non-Hispanic black, Mexican American, or other);

Model 2: further adjusted for BMI (<25, 25-30, ≥30 kg/m^2^), education level (less than high school, high school or equivalent, or college or above), family income-poverty ratio (0-1.0, 1.0-3.0, or >3.0), smoking status (never smoker, current smoker, or former smoker), drinking status (non-drinker, low-to-moderate drinker, heavy drinker, or former drinker), duration of diabetes (≤3, 3-10, or >10 years), diabetic medication use (none, only oral medication, insulin, or others), HbA1c (<7%, or ≥7%), hypertension, hyperlipidemia, ASCVD, CKD (yes, or no).
